# Knowledge of ovarian cancer symptoms among women in Palestine: a national cross-sectional study

**DOI:** 10.1186/s12889-021-12044-5

**Published:** 2021-11-03

**Authors:** Mohamedraed Elshami, Areej Yaseen, Mohammed Alser, Ibrahim Al-Slaibi, Hadeel Jabr, Sara Ubaiat, Aya Tuffaha, Salma Khader, Reem Khraishi, Inas Jaber, Zeina Abu Arafeh, Sondos Al-Madhoun, Aya Alqattaa, Asmaa Abd El Hadi, Ola Barhoush, Maysun Hijazy, Tamara Eleyan, Amany Alser, Amal Abu Hziema, Amany Shatat, Falasteen Almakhtoob, Balqees Mohamad, Walaa Farhat, Yasmeen Abuamra, Hanaa Mousa, Reem Adawi, Alaa Musallam, Nasser Abu-El-Noor, Bettina Bottcher

**Affiliations:** 1 Harvard Medical School, 25 Shattuck Street, Boston, MA 02115 USA; 2 Ministry of Health, Gaza, Palestine; 3 Faculty of Medicine, Al-Quds University, Jerusalem, Palestine; 4 Almakassed Hospital, Jerusalem, Palestine; 5 Faculty of Medicine, Islamic University of Gaza, Gaza, Palestine; 6 Faculty of Medicine, Al-Quds University, Bethlehem, Palestine; 7 Faculty of medicine, An-Najah National University, Nablus, Palestine; 8 Faculty of Medicine, Al-Azhar University-Gaza, Gaza, Palestine; 9 Al-shiffa Hospital, Gaza, Palestine; 10 Faculty of Medicine, Palestine Polytechnic University, Hebron, Palestine; 11 Beit Jala Governmental Hospital, Bethlehem, Palestine; 12 Faculty of Medicine, Al-Quds University, Jenin, Palestine; 13 Al-Aqsa Hospital, Deir Albalah, Palestine; 14 Faculty of Nursing, Islamic University of Gaza, Gaza, Palestine

**Keywords:** Ovarian cancer, Early detection, Survival, Symptom, Awareness, Knowledge, Early presentation, Palestine

## Abstract

**Introduction:**

Ovarian cancer (OC) is often diagnosed at advanced stages. This study aimed to assess the Palestinian women’s knowledge about OC symptoms and determine the factors associated with having good knowledge.

**Methods:**

A cross-sectional study was conducted from July 2019 to March 2020 in the two main areas of Palestine: the West Bank and Jerusalem as well as the Gaza Strip. A translated-into-Arabic version of the validated OC awareness measure (OCAM) was utilized for data collection. Stratified convenience sampling was used to recruit adult women attending hospitals, primary healthcare centers, and public spaces at 11 governorates. The knowledge level was categorized into three categories based on the number of symptoms recognized: poor (0 to 4), fair (5 to 8), and good (9 to 11).

**Results:**

Of 6095 approached, 5618 participants completed the Arabic OCAM (response rate = 92.1%).A total of 5411 questionnaires were included in the analysis: 2278 from the Gaza Strip and 3133 from the West Bank and Jerusalem. Participants living in the West Bank and Jerusalem were older, of higher monthly income, and with more chronic diseases than those living in the Gaza Strip.

The most frequently identified symptoms were ‘extreme generalized fatigue’ (*n* = 3821, 70.6%), ‘unexplained weight loss’ (*n* = 3607, 66.7%), and ‘increased abdominal size on most days’ (*n* = 3252, 60.1%). On the other hand, the least recognized symptoms were ‘feeling full persistently’ (*n* = 1553, 28.7%) and ‘difficulty eating on most days’ (*n* = 1971, 36.4%).

Only 943 participants (17.4%) displayed good knowledge of OC symptoms. Participants from the Gaza Strip had a higher likelihood than participants from the West Bank and Jerusalem to have a good level of knowledge (21.0% vs. 14.8%). Being married, knowing someone with cancer, and visiting hospitals were all associated with a higher likelihood of having good knowledge level. However, living in the West Bank and Jerusalem was associated with a lower likelihood of having good knowledge.

**Conclusion:**

The overall knowledge of OC symptoms in this study was low. Educational interventions are needed to improve Palestinian women’s knowledge about OC symptoms.

**Supplementary Information:**

The online version contains supplementary material available at 10.1186/s12889-021-12044-5.

## Introduction

Ovarian cancer (OC) is the third most common gynecological malignancy worldwide with 313,959 new cases diagnosed in 2020. OC has the second highest number of deaths related to gynecological cancers with estimated 207,252 deaths in 2020 [1]. In the Arab countries, the incidence of OC ranges from as low as 0.9 per 100,000 women in Saudi Arabia to 8.0 per 100,000 women per year in Sudan [2]. In Palestine, OC is the second most common gynecological cancer after corpus uterine cancers. In 2020, the number of deaths related to OC in Palestine was 53 which was high compared with the 74 newly diagnosed cases [3]. This necessitates finding possible ways to reduce this mortality.

Being diagnosed at early stages of OC (stage I and II) has been shown to be associated with a higher 5-year survival rate than being diagnosed with advanced stages (stage III and IV) [4–6]. Previous studies showed that diagnosis at advanced stages is a multifactorial process; could be attributed in part to the late presentation, because of the low awareness of OC symptoms, barriers to women accessing the healthcare system, and lack of knowledge amongst primary care professionals [7, 8]. In addition, OC symptoms have a non-distinctive nature such as early satiety, lower back pain, pelvic pain, bloating, and abdominal pain [9]. Misinterpretation of these symptoms by women and their doctors may lead to delayed presentation and diagnosis [10].

The lack of effective screening programs in combination with the non-specific symptoms raise the importance of awareness about OC and its possible symptoms among women. High awareness could help to reduce morbidity and mortality rates associated with delayed treatment of OC especially in low-resource settings [11–14].

In order to gain more in-depth information about the OC knowledge status in Palestine, this national study aimed to: (i) evaluate the Palestinian women’s level of knowledge of OC symptoms, (ii) compare this knowledge among women living in the Gaza Strip as well as West Bank and Jerusalem (WBJ) given the different sociodemographic factors [3, 15–17], and (iii) determine the factors associated with good knowledge of OC symptoms.

## Materials and methods

### Study design and population

A national cross-sectional study was conducted from July 16th, 2019 to March 29th, 2020. In Palestine, women represent 49.1% of the total population with 51.7% of them aged 20 or older [3]. There are 16 governorates across Palestine distributed in the two main regions: the WBJ and the Gaza Strip. The Palestinian Ministry of Health (MoH) has 15 hospitals in the West Bank, 13 hospitals in the Gaza Strip, and no hospitals in Jerusalem. The Palestinian MoH also has 397 primary healthcare centers (PHCs) in the West Bank, 51 PHCs in the Gaza Strip, and 27 PHCs in Jerusalem [3]. Recruiting participants from these sites can cover most of the adult female population and may produce a representative sample from across Palestine [11–14].

Inclusion criteria for study hospitals were being a general (i.e., secondary) hospital and having a bed capacity of more than 100. Out of the 28 MoH hospitals, there were six eligible hospitals in the West Bank and five in the Gaza Strip. The non-governmental organizations had two eligible hospitals in Jerusalem. The PHCs were eligible to participate if they were classified as level four (i.e., providing all primary healthcare services). Among the 475 PHCs, 26 were classified as level four; 17 in the WBJ and nine in the Gaza Strip [3]. Public places of the corresponding governorates were eligible. These included malls, markets, parks, public squares, mosques, churches, bus stops, parking lots and others.

Inclusion criteria of study participants were being an adult female (≥ 18 years), having the Palestinian nationality, and visiting a place or being admitted to one of the participating sites. Exclusion criteria were having a nationality other than the Palestinian, working or studying in the medical field, being a visitor or a patient in the oncology departments, and being unable to complete the questionnaire.

### Sampling methods

A stratified convenience sampling was used to recruit participants from 12 hospitals (seven in the WBJ and five in the Gaza Strip), 10 PHCs (six in the WBJ and four in the Gaza Strip), and public spaces of 11 governorates (seven in the WBJ and four in the Gaza Strip). The wide coverage from different places was intended to create a diverse study cohort that could represent the Palestinian community appropriately. In 2019, the estimated female population aged 15 years or older was 947,100 females in the WBJ and 587,271 females in the Gaza Strip [18]. Based on the sample size calculator of the Australian Bureau of Statistics [19], with a confidence level of 95.0% and a margin of error of 3.0%, the calculated minimum sample size was 1066 for each of the WBJ and the Gaza Strip. A total minimum sample size of 2132 was needed.

### Questionnaire and data collection

As an assessment tool, a modified version of the ovarian cancer awareness measure (OCAM) was used [20]. The questionnaire consisted of three sections. The first section described the socio-demographic data. The second section assessed the confidence of participants about their ability to recognize OC symptoms. The third section evaluated women’s awareness of the OC symptoms. A 4-point scale (1 = not at all confident, 4 = very confident) was used to assess the confidence to detect OC symptoms, whereas a 5-point Likert scale (1 = strongly disagree, 5- strongly agree) was used to evaluate the participants’ knowledge of 11 OC symptoms.

The OCAM was translated from English to Arabic by two bilingual health care professionals who are involved in health-related research and then back-translated into English by another two bilingual researchers. Content validity was then examined by having the questionnaire reviewed by a panel of five experts in the fields of gynecology, oncology, and public health. Face validity was also examined by conducting a pilot study with 128 respondents who met the inclusion criteria to test the clarity of the questions of the Arabic version. Those who had been included in the pilot test were excluded from the study. Internal consistency was evaluated using Cronbach’s Alpha, which reached an acceptable value (α = 0.778).

The OCAM was modified for the purposes of this study. To minimize the possibility that participants will answer questions randomly, the original questions with yes/no/unknown responses were modified into a 5-point Likert scale (1 = strongly disagree, 5- strongly agree). The participants’ responses were then converted to correct/incorrect responses similar to what was done in previous studies [13, 14]. In addition, ‘unexplained weight loss’ was added to the list of OC symptoms as it was used in other forms of the cancer awareness measure tool [21, 22] and it was thought to be helpful to include it in the context of OC.

Adult women who attended the included hospitals, PHCs or public spaces during the study period were invited to participate in the study. Women were invited to complete the Arabic OCAM in face-to-face interviews. The data collection tool utilized was *‘Kobo Toolbox’*, a secure, user-friendly, and smartphones usable tool [23]. Female data collectors were trained on how to approach the participants, explain the study and questionnaire, and use the electronic tool. The choice of female data collectors was intended to facilitate communication with study participants and to make the participants feel more comfortable talking to females on this sensitive subject.

### Statistical analysis

Participant characteristics were summarized using descriptive statistics. The median and interquartile range (IQR) were used to describe continuous variables that were non-normally distributed. Frequencies and percentages were used to describe categorical variables. Age was categorized into two groups to reflect the age-related risk of OC (45 years or older) [20]. Menarche was categorized into three categories to describe early (≤ 10 years), normal (11–15 years), and late (≥ 16 years) occurrence [24]. The cutoff of 1450 NIS (about $450) was chosen to divide the monthly income into two groups as it was the minimum wage in Palestine [15].

A baseline comparison between the WBJ vs. the Gaza Strip was performed using Kruskal-Wallis test if the variable was continuous or using Pearson’s Chi-square test if it was categorical.

The participants’ confidence to detect possible OC symptoms was described by frequencies and percentages with a comparison between the WBJ vs. the Gaza Strip being performed using Pearson’s Chi-Square test. For questions asking about OC symptoms, responding with ‘strongly agree’ or ‘agree’ were considered as a correct answer, while responding with ‘strongly disagree’, ‘disagree’, or ‘not sure’ were considered as an incorrect answer. OC symptoms were categorized into two categories: symptoms with pain and other symptoms. Recognition of each of the OC symptoms was described using frequencies and percentages with comparisons being made using Pearson’s Chi-Square test. This was followed by running bivariable and multivariable logistic regression analyses. Results of the bivariable analyses are provided in the supplementary materials. Please see additional file 1. The model of the multivariable analysis adjusted for age-group, menarche, educational level, occupation, monthly income, residency, having a chronic disease, knowing someone with cancer, marital status, and site of data collection. This model was pre-specified based on the existing literature [4, 25–28].

In order to assess the participant’s level of knowledge about OC symptoms, a scoring system used in previous studies was utilized in this study [13, 29]. For each correctly recognized OC symptom, one point was given. The total score (ranging from 0 to 11) was calculated and categorized into three categories based on the number of symptoms recognized: poor knowledge (0 to 4), fair knowledge (5 to 8), and good knowledge (9 to 11). Pearson’s Chi-Square test was used to make comparisons in the level of knowledge between the participants from the WBJ vs. the Gaza Strip. Bivariable and multivariable logistic regression was used to test the association of participant characteristics with having a good level of knowledge.

Missing data were completely random and were unrelated to the study variables. Complete responses were included in the final analysis (i.e., complete case analysis). Data were analyzed using Stata software version 16.0 (StataCorp, College Station, Texas, United States).

## Results

### Participant characteristics

Of 6095 approached, 5618 participants completed the questionnaire (response rate = 92.1%). A total of 5411 questionnaires were included in the analysis (49 were excluded and 158 had missing data): 3133 from the WBJ and 2278 from the Gaza Strip. The median age [IQR] for all participants was 32.0 years [24.0, 44.0] (Table 1). Participants living in the WBJ were older, of higher monthly income, and with more chronic diseases than those living in the Gaza Strip.

**Table 1:** Characteristics of study participants

Characteristic	Total (***n*** = 5411)	Gaza Strip (***n*** = 2278)	WBJ (***n*** = 3133)	***p***-value
**Age**, median [IQR]	32 [24, 44]	30 [24, 40]	34 [25, 46]	< 0.001
**Age group**, n (%)				
18 to 44	4151 (76.7)	1872 (82.2)	2279 (72.2)	< 0.001
45 or older	1260 (23.3)	406 (17.8)	854 (27.3)	
**Menarche**, n (%)				
Early (≤ 10 years)	65 (1.2)	20 (0.9)	45 (1.4)	< 0.001
Normal (11–15 years)	4658 (86.1)	1923 (84.4)	2735 (87.3)	
Late (≥ 16 years)	688 (12.7)	335 (14.7)	353 (11.3)	
**Educational level**, n (%)				
Illiterate	76 (1.4)	20 (0.9)	56 (1.8)	< 0.001
Primary	321 (5.9)	89 (3.9)	232 (7.4)	
Preparatory	786 (14.5)	313 (13.7)	473 (15.1)	
Secondary	1833 (33.9)	908 (39.9)	925 (29.5)	
Diploma	586 (10.8)	236 (10.4)	350 (11.2)	
Bachelor	1723 (31.8)	692 (30.3)	1031 (32.9)	
Postgraduate	86 (1.7)	20 (0.9)	66 (2.1)	
**Occupation**, n (%)				
Housewife	3671 (67.8)	1837 (80.6)	1834 (58.5)	< 0.001
Employed	1095 (20.2)	254 (11.2)	841 (26.8)	
Retired	47 (0.9)	9 (0.4)	38 (1.2)	
Student	598 (11.1)	178 (7.8)	420 (13.5)	
**Monthly income ≥ 1450 NIS**, n (%)	3330 (61.5)	474 (20.8)	2856 (91.2)	< 0.001
**Having a chronic disease**, n (%)	1097 (20.3)	350 (15.4)	747 (23.8)	< 0.001
**Knowing someone with cancer**, n (%)	2746 (50.8)	1104 (48.5)	1642 (52.4)	0.016
**Marital status**, n (%)				
Single	1248 (23.1)	374 (16.4)	874 (27.9)	< 0.001
Married	3952 (73.0)	1836 (80.6)	2116 (67.5)	
Divorced	88 (1.6)	33 (1.4)	55 (1.8)	
Widowed	123 (2.3)	35 (1.6)	88 (2.8)	
**Site of data collection**, n (%)				
Public Spaces	1645 (30.4)	596 (26.2)	1049 (33.5)	< 0.001
Hospitals	1735 (32.1)	650 (28.5)	1085 (34.6)	
Primary healthcare centers	2031 (37.5)	1032 (45.3)	999 (31.9)	

*n* number of participants, *IQR* interquartile range, *WBJ* West Bank and Jerusalem

### Confidence and recognition of OC symptoms

Only 1129 women (20.9%) felt confident to notice a possible OC symptom. Women from the Gaza Strip were more likely to be confident than women from the WBJ (30.5% vs 13.9%). The most frequently identified symptoms were ‘extreme generalized fatigue’ (*n* = 3821, 70.6%), ‘unexplained weight loss’ (*n* = 3607, 66.7%), and ‘increased abdominal size on most days’ (*n* = 3252, 60.1%) (Table 2). These symptoms were also the most identified symptoms in both the WBJ and the Gaza Strip. The least recognized symptoms were ‘feeling full persistently’ (*n* = 1553, 28.7%) and ‘difficulty eating on most days’ (*n* = 1971, 36.4%). These symptoms were also the least identified symptoms in both the WBJ and the Gaza Strip.

**Table 2:** Recognition of ovarian cancer symptoms

Category of Symptom	Symptom	Total (***n*** = 5411) n (%)	Gaza Strip (***n*** = 2278) n (%)	WBJ (***n*** = 3133) n (%)	***p***-value
**Symptoms with pain**	Persistent low back pain	3096 (57.2)	1342 (58.9)	1754 (56.0)	0.032
	Persistent pain in the pelvis	2799 (51.7)	1125 (49.4)	1674 (53.4)	0.003
	Persistent pain in the abdomen	2617 (48.4)	1079 (47.4)	1538 (49.1)	0.21
**Other symptoms**	Extreme generalized fatigue	3821 (70.6)	1621 (71.2)	2200 (70.2)	0.45
	Unexplained weight loss	3607 (66.7)	1552 (68.1)	2055 (65.6)	0.051
	Increased abdominal size on most days	3252 (60.1)	1424 (62.5)	1828 (58.3)	0.002
	Passing more urine than usual	2614 (48.3)	1136 (49.9)	1478 (47.2)	0.051
	Changes in bowel habit	2206 (40.8)	930 (40.8)	1276 (40.7)	0.94
	Persistent bloating	2166 (40.0)	981 (43.1)	1185 (37.8)	< 0.001
	Difficulty eating on most days	1971 (36.4)	932 (40.9)	1039 (33.2)	< 0.001
	Feeling full persistently	1553 (28.7)	754 (33.1)	799 (25.5)	< 0.001

*n* number of participants, *IQR* interquartile range, *WBJ* West Bank and Jerusalem

### Good knowledge and its associated factors

Only 943 participants (17.4%) displayed good knowledge (prompt recognition of more than 8 out of 11 OC symptoms) (Table 3). Participants from the Gaza Strip had a higher likelihood than participants from the WBJ to have a good level of knowledge (21.0% vs. 14.8%). On the multivariable analysis, being married, knowing someone with cancer, and visiting hospitals were all associated with an increase in the odds of having a good knowledge level of OC symptoms (Table 4). However, living in the WBJ was associated with a decrease in the odd of having good knowledge.

**Table 3:** Knowledge level of ovarian cancer symptoms among study participants

Level	Total n (%)	Gaza Strip n (%)	WBJ n (%)	***p***-value
**Poor**	1943 (35.9)	780 (34.2)	1163 (37.1)	< 0.001
**Fair**	2525 (46.7)	1020 (44.8)	1505 (48.0)	
**Good**	943 (17.4)	478 (21.0)	465 (14.8)	

*n* number of participants, *WBJ* West Bank and Jerusalem

**Table 4:** Association between having a good knowledge of ovarian cancer symptoms and participant characteristics

Characteristic	Good knowledge			
	COR (95% CI)	***p***-value	AOR (95% CI)*	***p***-value
**Age group**				
18 to 44	Ref	Ref	Ref	Ref
45 or older	1.25 (1.07–1.47)	0.006	1.14 (0.94–1.38)	0.19
**Menarche**				
Normal (11–15 years)	Ref	Ref	Ref	Ref
Early (≤ 10 years)	1.35 (0.76–2.45)	0.33	1.35 (0.74–2.49)	0.33
Late (≥ 16 years)	1.27 (1.04–1.55)	0.021	1.20 (0.98–1.47)	0.08
**Educational level**				
Illiterate	Ref	Ref	Ref	Ref
Primary	2.05 (1.01–4.18)	0.049	1.89 (0.92–3.88)	0.08
Preparatory	1.75 (0.88–3.49)	0.11	1.62 (0.81–3.26)	0.17
Secondary	1.51 (0.77–2.97)	0.23	1.61 (0.81–3.21)	0.18
Diploma	1.03 (0.51–2.08)	0.94	1.26 (0.61–2.60)	0.53
Bachelor	1.16 (0.59–2.29)	0.66	1.55 (0.77–3.12)	0.22
Postgraduate	1.18 (0.48–2.86)	0.72	1.68 (0.67–4.21)	0.27
**Occupation**				
Housewife	Ref	Ref	Ref	Ref
Employed	0.64 (0.53–0.77)	< 0.001	0.87 (0.70–1.09)	0.24
Retired	0.48 (0.19–1.22)	0.13	0.58 (0.22–1.50)	0.26
Student	0.48 (0.36–0.63)	< 0.001	0.77 (0.55–1.07)	0.12
**Monthly income**				
< 1450 NIS	Ref	Ref	Ref	Ref
≥ 1450 NIS	0.64 (0.56–0.74)	< 0.001	0.84 (0.68–1.04)	0.10
**Residency**				
Gaza Strip	Ref	Ref	Ref	Ref
WBJ	0.66 (0.57–0.76)	< 0.001	0.75 (0.61–0.93)	0.008
**Having a chronic disease**				
No	Ref	Ref	Ref	Ref
Yes	1.20 (1.01–1.42)	0.034	1.03 (0.85–1.25)	0.76
**Knowing someone with cancer**				
No	Ref	Ref	Ref	Ref
Yes	1.44 (1.25–1.66)	< 0.001	1.43 (1.24–1.65)	< 0.001
**Marital status**				
Single	Ref	Ref	Ref	Ref
Married	1.82 (1.50–2.20)	< 0.001	1.30 (1.03–1.65)	0.028
Divorced	1.44 (0.79–2.61)	0.23	1.18 (0.64–2.19)	0.59
Widowed	1.66 (1.01–2.71)	0.045	1.05 (0.61–1.79)	0.86
**Site of data collection**				
Public Spaces	Ref	Ref	Ref	Ref
Hospitals	1.66 (1.39–1.99)	< 0.001	1.46 (1.20–1.76)	< 0.001
Primary healthcare centers	1.17 (0.98–1.41)	0.09	1.05 (0.87–1.28)	0.59

*COR* crude odds ratio, *AOR* adjusted odds ratio, *CI* confidence interval, *WBJ* West Bank and Jerusalem

* Adjusted for age-group, menarche, educational level, occupation, monthly income, marital status, residency, having a chronic disease, knowing someone with cancer, and stratum

### Association between recognizing the three most identified symptoms and participant characteristics

Having a bachelor’s degree, knowing someone with cancer, and visiting hospitals were all associated with an increase in the odds of recognizing the three most identified symptoms. Supplementary Table 1 summarizes the association between the other participant characteristics and the recognition of the three most identified OC symptoms.

### Association between recognizing symptoms with pain and participant characteristics

On the multivariable analysis, women who had a postgraduate degree were more likely than illiterate women to identify all symptoms with pain (supplementary Table 2). In addition, women who knew someone with cancer had a higher likelihood than women who did not to identify all these symptoms with pain. On the other hand, participants recruited from PHCs were less likely than participants recruited from public spaces to recognize all symptoms with pain.

### Association between recognizing other OC symptoms and participant characteristics

Knowing someone with cancer was associated with an increase in the odds of recognizing all other OC symptoms. Supplementary Table 3 summarizes the association between participant characteristics and the recognition of all other OC symptoms.

## Discussion

There has been a substantial need to measure the awareness of OC symptoms among Palestinian women. Promoting this awareness is associated with a shorter interval to visit the doctor and having an early diagnosis, hence a higher survival rate. The results of this study showed that only 17.4% of the participants demonstrated good knowledge of OC symptoms, defined as recognition of more than eight of the 11 potential OC symptoms. Participants from the Gaza Strip were more likely to have higher knowledge levels than participants from the WBJ. The factors associated with having good knowledge were being married, knowing someone with cancer, and visiting hospitals. The most frequently identified symptom was ‘extreme generalized fatigue’ followed by ‘unexplained weight loss’ and ‘increased abdominal size on most days’. The least recognized symptoms were ‘feeling full persistently’ and ‘difficulty eating on most days’.

### Knowledge level of OC symptoms

Awareness of OC symptoms is a key determinant in seeking early medical attention and could lead to a better prognosis [28]. Therefore, there has been a growing interest to focus on increasing the level of women’s knowledge of OC symptoms in order to improve earlier diagnosis [30]. In concordance with previous studies from other countries, this study found poor knowledge of OC symptoms among Palestinian women [4, 25–27].

Puckett and colleagues demonstrated an increase in knowledge of OC symptoms following a targeted campaign using specifically developed educational materials. In addition, the authors found that the confidence of women to discuss gynecological cancers and their symptoms also increased [28]. Similar educational interventions are needed, especially in low-resource settings as in Palestine, to promote recognition of symptoms and facilitate early presentation. However, establishing these campaigns requires sustained funding and significant ongoing efforts, which often prohibits their implementation in low and middle-income countries such as Palestine. Nevertheless, reduced morbidity and mortality of OC due to earlier presentation could contribute to long-term cost-effectiveness. Moreover, integration of such education in school curricula or at visits to sexual and reproductive health clinics or other available women’s health touch points such as breast and cervical screening might facilitate such public education.

This study found that the least recognized symptoms were ‘feeling full persistently’ and ‘difficulty eating on most days’. Similar results were reported in previous studies [25, 26, 31]. Whereas all these symptoms might be present in both malignant and benign ovarian conditions, these two may be regarded as more general symptoms or even be associated with gastrointestinal disorders, which may explain why they were less likely to be recognized as OC symptoms. It might be helpful for campaigns aiming to improve OC awareness to consider enlightening women on distinguishing the different characteristics of OC symptoms. For example, women who experience non-specific symptoms related to OC tend to have more frequent and severe symptoms, compared with those who have the same symptoms due to benign conditions [9].

### Comparing knowledge of OC symptoms between the Gaza Strip vs. the WBJ

In this study, participants living in the Gaza Strip were more likely to have a good knowledge level of OC symptoms than participants living in the WBJ. Several factors may explain this. Firstly, the economic situation could be an important determinant of the place to receive treatment if needed. The economic situation in the Gaza Strip is poorer than that in the WBJ [17]. This may drive women in the Gaza Strip to rely solely on using their health insurance to visit governmental hospitals to get their treatment, where they could also learn more about OC symptoms. This is also supported by our finding that visiting hospitals was shown to be an important factor to have a good knowledge of OC symptoms. Secondly, the female population in the Gaza Strip is younger than the female population in the WBJ [3] which was also found in this study. A previous report showed that young women (≤ 44 years) were using social media platforms more often than the older women in Palestine [32]. The report also found a higher proportion of social media users in the Gaza Strip than in the WBJ. It seems that young women in the Gaza Strip more often use social media to reach various health pages and learn more about OC symptoms than women in the WBJ. Finally, the importance of the extended family and social life within the Palestinian community might open opportunities for exchange of information and knowledge [33–35]. This might be more common in the Gaza Strip than in the WBJ due to the more traditional life and paucity of opportunities in the Gaza Strip compared with the WBJ. However, further research is needed to delineate the resources of information that Palestinian women use to build their knowledge.

Recognition of cancer symptoms is not the only contributor to the delayed time from first appearance of a symptom to the diagnosis of the disease [36–38]. Various contributors are known to impact the length of this interval, including access to healthcare facilities, quality of health services and barriers to presenting to a healthcare professional, which can include emotional, practical and service barriers [14, 39]. A previous study in the Gaza Strip investigating potential barriers to early presentation with suspected cancer warning signs found emotional barriers such as fear and worry widespread among the study participants. However, better knowledge of cancer warning signs was associated with lower barriers to see a healthcare professional [14]. Thus, better knowledge might reduce the time to presentation by mitigating these barriers [40, 41].

In low- and middle-income countries, such as Palestine, the impact of late presentation on cancer survival was found to be greater than in high-income countries [36, 37, 42]. Therefore, increasing the public awareness of cancer warning signs might be one contributor to reducing the interval from recognizing a possible cancer symptom to diagnosis [43–45].

### Future directions

The low level of knowledge of OC symptoms among Palestinian women opens the prospects to include OC in national educational campaigns beside other cancers. These campaigns should also be tailored to match the understanding of women from different backgrounds. In addition, awareness campaigns should focus on improving women’s ability to distinguish the different characteristics (i.e., persistence and frequency) of OC symptoms. This could be especially helpful for recognizing symptoms of a non-specific nature.

Previous reports showed that about 80.0% of the households in Palestine have internet access at home (84.8% in the WBJ and 73.0% in the Gaza Strip) [46]. The reports also showed that the percentage of households that own one Smartphone or more was 86.0% in Palestine (91.0% in the WBJ and 78.0% in the Gaza Strip) [47]. To overcome the financial barrier of implementing on-site campaigns and given the growing use of social media, these platforms could be utilized as a vehicle to deliver information to the Palestinian women. This may also help in raising the outreach to women who are not able to visit sites of campaigns for different emotional and practical reasons. On the other hand, women living in areas with digital deprivation in Palestine could be targeted by local media or text messages. The latter could be feasible with the wide availability of cellular mobile lines across Palestine [47].

### Strengths and limitations

The main strengths of this study were the high response rate and the large sample size covering most areas of Palestine. This study had some limitations though. Firstly, it assessed the recognition of OC symptoms while it might have been helpful to evaluate the recall of these symptoms as well. Secondly, although the current study recruited a large number of participants from different backgrounds and places across Palestine, the use of stratified convenience sampling might still be a limitation as it makes it challenging to guarantee the generation of a representative sample. Thirdly, the small number of women aged 65 and over means that only few women at the highest risk of OC were included in the study. However, the greater inclusion of younger women may allow repeated exposure (i.e., education) over time that builds awareness of OC symptoms. Finally, visitors to healthcare facilities were invited to participate in this study. This might have led to recruitment of people displaying a presumably better knowledge. However, the inclusion of women attending public spaces may have mitigated this.

## Conclusions

The knowledge level of OC symptoms among women included in this study was low with only 17.4% showing good knowledge. Participants from the Gaza Strip were more likely to demonstrate higher knowledge than participants from the WBJ. The most frequently recognized symptom was ‘extreme generalized fatigue’ followed by ‘unexplained weight loss’ and ‘increased abdominal size on most days’. The least recognized symptoms were ‘feeling full persistently’ and ‘difficulty eating on most days’. Being married, knowing someone with cancer, and visiting hospitals were all associated with an increase in the likelihood of having good knowledge of OC symptoms. Targeted education campaigns are needed to improve OC symptom knowledge and, thus, improve early diagnosis and survival of OC patients.

## Supplementary Information

**Additional file 1:** **Supplementary Table 1.** Multivariable logistic regression analyzing the association between recognizing the three most identified symptoms of ovarian cancer and participant characteristics. **Supplementary Table 2.** Multivariable logistic regression analyzing the association between recognizing ovarian cancer symptoms with pain and participant characteristics. **Supplementary Table 3.** Multivariable logistic regression analyzing the association between recognizing other ovarian cancer symptoms and participant characteristics. **Supplementary Table 4.** Bivariable logistic regression analyzing the association between recognizing the three most identified symptoms of ovarian cancer and participant characteristics. **Supplementary Table 5.** Bivariable logistic regression analyzing the association between recognizing ovarian cancer symptoms with pain and participant characteristics. **Supplementary Table 6.** Bivariable logistic regression analyzing the association between recognizing other ovarian cancer symptoms and participant characteristics.

## Data Availability

The dataset used and analyzed during the current study is available from the corresponding author on reasonable request.

## Abbreviations

OC: ovarian cancer
WBJ: West Bank and Jerusalem
PHCs: primary healthcare centers
MoH: ministry of health
OCAM: ovarian cancer awareness measure
CI: confidence interval
OR: odds ratio
