# Supplementary material for: Knowledge of ovarian cancer symptoms among women in Palestine: a national cross-sectional study
Source: BMC Public Health. 2021 Nov 3;21:1992. doi: 10.1186/s12889-021-12044-5 (PMC8567700; doi:10.1186/s12889-021-12044-5)
Supplement: Supplementary file 1 — Additional file 1: Supplementary Table 1. Multivariable logistic regression analyzing the association between recognizing the three most identified symptoms of ovarian cancer and participant characteristics. Supplementary Table 2. Multivariable logistic regression analyzing the association between recognizing ovarian cancer symptoms with pain and participant characteristics. Supplementary Table 3. Multivariable logistic regression analyzing the association between recognizing other ovarian cancer symptoms and participant characteristics. Supplementary Table 4. Bivariable logistic regression analyzing the association between recognizing the three most identified symptoms of ovarian cancer and participant characteristics. Supplementary Table 5. Bivariable logistic regression analyzing the association between recognizing ovarian cancer symptoms with pain and participant characteristics. Supplementary Table 6. Bivariable logistic regression analyzing the association between recognizing other ovarian cancer symptoms and participant characteristics. [file 12889_2021_12044_MOESM1_ESM.docx]

**Knowledge of Ovarian Cancer Symptoms among Women in Palestine: A National Cross- sectional Study**

Mohamedraed Elshami, MD, MMSc^1,2^*, Areej Yaseen^3^*, Mohammed Alser, MD^2^*, Ibrahim Al-Slaibi, MD^4^*, Hadeel Jabr, MD^5^, Sara Ubaiat^6^, Aya Tuffaha^7^, Salma Khader^3^, Reem Khraishi^7^, Inas Jaber^3^, Zeina Abu Arafeh^3^, Sondos Al-Madhoun^8^, Aya Alqattaa^5^, Asmaa Abd El Hadi^5^, Ola Barhoush^3^, Maysun Hijazy^5^, Tamara Eleyan^3^, Amany Alser^9^, Amal Abu Hziema^5^, Amany Shatat^5^, Falasteen Almakhtoob^10^, Balqees Mohamad, MD^11^, Walaa Farhat^12^, Yasmeen Abuamra^8^, Hanaa Mousa^5^, Reem Adawi^3^, Alaa Musallam, MD^13^, Nasser Abu-El-Noor, PhD^14#^, Bettina Bottcher, MD, PhD^5#^

*Contributed equally as a first co-author.

^#^Contributed equally as a senior co-author.

^1^Harvard Medical School, Boston, MA, USA.
^2^Ministry of Health, Gaza, Palestine.

^3^Faculty of Medicine, Al-Quds University, Jerusalem, Palestine.

^4^Almakassed Hospital, Jerusalem, Palestine.

^5^Faculty of Medicine, Islamic University of Gaza, Gaza, Palestine.

**^6^**Faculty of Medicine, Al-Quds University, Bethlehem, Palestine.

^7^Faculty of medicine, An-Najah National University, Nablus, Palestine.

^8^Faculty of Medicine, Al-Azhar university-Gaza, Gaza, Palestine.
^9^Al-shiffa Hospital, Gaza, Palestine.
^10^Faculty of Medicine, Palestine Polytechnic University, Hebron, Palestine.
^11^Beit Jala Governmental Hospital, Bethlehem, Palestine.
^12^Faculty of Medicine, Al-Quds University, Jenin, Palestine.

^13^Al-Aqsa Hospital, Deir Albalah, Palestine.

^14^Faculty of Nursing, Islamic University of Gaza, Gaza, Palestine.

**Corresponding author**

Mohamedraed Elshami, MD, MMSc

Harvard Medical School, 25 Shattuck Street, Boston, Massachusetts, USA 02115

Phone: 832-245-6055

Email: mohamedraed.elshami@gmail.com

Supplementary table 1: Multivariable logistic regression analyzing the association between recognizing the three most identified symptoms of ovarian cancer and participant characteristics.

| **Characteristic** | **Extreme generalized fatigue** | | **Unexplained weight loss** | | **Increased abdominal size on most days** | |
| --- | --- | --- | --- | --- | --- | --- |
|  | **AOR (95% CI)*** | **p-value** | **AOR (95% CI)*** | **p-value** | **AOR (95% CI)*** | **p-value** |
| **Age group**  18 to 44  45 or older | Ref  0.89 (0.75- 1.06) | Ref  0.19 | Ref  0.96 (0.81- 1.14) | Ref  0.64 | Ref  1.00 (0.86- 1.17) | Ref  0.97 |
| **Menarche**  Normal (11-15 years)  Early (≤ 10 years)  Late (≥ 16 years) | Ref  0.62 (0.37- 1.04)  0.99 (0.82- 1.18) | Ref  0.07  0.90 | Ref  0.81 (0.48- 1.37)  1.04 (0.87- 1.24) | Ref  0.43  0.64 | Ref  0.74 (0.45- 1.23)  1.07 (0.91- 1.27) | Ref  0.25  0.41 |
| **Educational level**  Illiterate  Primary  Preparatory  Secondary  Diploma  Bachelor  Postgraduate | Ref  1.34 (0.80- 2.25)  1.99 (1.22- 3.26)  2.07 (1.27- 3.35)  2.31 (1.39- 3.85)  2.92 (1.78- 4.79)  4.52 (2.20- 9.29) | Ref  0.27  0.006  0.003  0.001  <0.001  <0.001 | Ref  1.09 (0.65- 1.83)  1.42 (0.86- 2.33)  1.46 (0.90- 2.38)  1.56 (0.94- 2.60)  1.66 (1.01- 2.72)  2.24 (1.12- 4.44) | Ref  0.75  0.17  0.13  0.09  0.046  0.022 | Ref  1.11 (0.67- 1.85)  1.44 (0.89- 2.33)  1.74 (1.08- 2.80)  1.66 (1.01- 2.72)  1.95 (1.20- 3.16)  1.88 (0.98- 3.61) | Ref  0.68  0.14  0.023  0.046  0.007  0.06 |
| **Occupation**  Housewife  Employed  Retired  Student | Ref  0.74 (0.62- 0.89)  0.61 (0.32- 1.14)  1.20 (0.93- 1.53) | Ref  0.001  0.12  0.16 | Ref  0.86 (0.73- 1.03)  0.90 (0.47- 1.70)  0.90 (0.71- 1.14) | Ref  0.10  0.74  0.39 | Ref  0.93 (0.79- 1.10)  0.76 (0.41- 1.39)  1.17 (0.93- 1.47) | Ref  0.40  0.37  0.18 |
| **Monthly income**  < 1450 NIS  ≥ 1450 NIS | Ref  1.23 (1.03- 1.48) | Ref  0.026 | Ref  0.96 (0.80- 1.15) | Ref  0.66 | Ref  1.18 (0.99- 1.40) | Ref  0.06 |
| **Residency**  Gaza Strip  WBJ | Ref  0.83 (0.69- 0.99) | Ref  0.042 | Ref  0.91 (0.76- 1.08) | Ref  0.27 | Ref  0.74 (0.63- 0.87) | Ref  <0.001 |
| **Having a chronic disease**  No  Yes | Ref  1.26 (1.06- 1.49) | Ref  0.010 | Ref  1.01 (0.86- 1.19) | Ref  0.92 | Ref  1.06 (0.90- 1.23) | Ref  0.50 |
| **Knowing someone with cancer**  No  Yes | Ref  1.71 (1.52- 1.94) | Ref  <0.001 | Ref  1.69 (1.50- 1.90) | Ref  <0.001 | Ref  1.47 (1.31- 1.65) | Ref  <0.001 |
| **Marital status**  Single  Married  Divorced  Widowed | Ref  1.36 (1.14- 1.62)  1.21 (0.75- 1.96)  0.90 (0.59- 1.37) | Ref  0.001  0.43  0.62 | Ref  1.33 (1.12- 1.58)  1.12 (0.71- 1.79)  1.26 (0.82- 1.94) | Ref  0.001  0.62  0.29 | Ref  1.17 (0.99- 1.38)  0.89 (0.57- 1.39)  0.94 (0.63- 1.41) | Ref  0.07  0.60  0.77 |
| **Site of data collection**  Public spaces  Hospitals  Primary healthcare centers | Ref  1.48 (1.25- 1.75)  0.78 (0.67- 0.91) | Ref  <0.001  0.001 | Ref  1.53 (1.30- 1.79)  0.76 (0.66- 0.88) | Ref  <0.001  <0.001 | Ref  1.52 (1.31- 1.77)  0.92 (0.80- 1.06) | Ref  <0.001  0.24 |

AOR= adjusted odds ratio, CI= confidence interval, WBJ= West Bank and Jerusalem.
* Adjusted for age-group, menarche, educational level, occupation, monthly income, residency, having a chronic disease,
knowing someone with cancer, and site of data collection.

Supplementary table 2: Multivariable logistic regression analyzing the association between recognizing ovarian cancer symptoms with pain and participant characteristics.

| **Characteristic** | **Persistent low back pain** | | **Persistent pain in the pelvis** | | **Persistent pain in the abdomen** | |
| --- | --- | --- | --- | --- | --- | --- |
|  | **AOR (95% CI)*** | **p-value** | **AOR (95% CI)*** | **p-value** | **AOR (95% CI)*** | **p-value** |
| **Age group**  18 to 44  45 or older | Ref  1.01 (0.86- 1.18) | Ref  0.94 | Ref  1.08 (0.93- 1.26) | Ref  0.32 | Ref  1.09 (0.94- 1.27) | Ref  0.27 |
| **Menarche**  Normal (11-15 years)  Early (≤ 10 years)  Late (≥ 16 years) | Ref  0.77 (0.47- 1.27)  1.10 (0.93- 1.29) | Ref  0.31  0.28 | Ref  0.75 (0.46- 1.24)  0.92 (0.78- 1.08) | Ref  0.26  0.30 | Ref  1.05 (0.64- 1.72)  1.11 (0.94- 1.30) | Ref  0.86  0.22 |
| **Educational level**  Illiterate  Primary  Preparatory  Secondary  Diploma  Bachelor  Postgraduate | Ref  1.59 (0.94- 2.67)  1.76 (1.07- 2.90)  2.16 (1.32- 3.53)  2.14 (1.29- 3.57)  2.59 (1.57- 4.26)  2.74 (1.42- 5.30) | Ref  0.08  0.025  0.002  0.003  <0.001  0.003 | Ref  0.85 (0.51- 1.41)  0.93 (0.57- 1.50)  1.10 (0.68- 1.76)  1.17 (0.72- 1.92)  1.35 (0.84- 2.18)  1.99 (1.04- 3.84) | Ref  0.53  0.75  0.70  0.53  0.22  0.037 | Ref  1.11 (0.66- 1.86)  1.43 (0.88- 2.33)  1.56 (0.96- 2.53)  1.71 (1.03- 2.83)  1.85 (1.13- 3.02)  2.29 (1.20- 4.38) | Ref  0.69  0.15  0.07  0.037  0.014  0.012 |
| **Occupation**  Housewife  Employed  Retired  Student | Ref  0.90 (0.76- 1.06)  0.60 (0.32- 1.09)  1.27 (1.01- 1.59) | Ref  0.20  0.09  0.042 | Ref  0.95 (0.81- 1.12)  0.60 (0.33- 1.10)  1.61 (1.28- 2.02) | Ref  0.55  0.10  <0.001 | Ref  0.92 (0.78- 1.08)  0.46 (0.25- 0.87)  1.08 (0.87- 1.36) | Ref  0.29  0.016  0.47 |
| **Monthly income**  < 1450 NIS  ≥ 1450 NIS | Ref  1.19 (1.01- 1.40) | Ref  0.046 | Ref  1.16 (0.99- 1.37) | Ref  0.08 | Ref  0.96 (0.81- 1.13) | Ref  0.62 |
| **Residency**  Gaza Strip  WBJ | Ref  0.78 (0.66- 0.92) | Ref  0.003 | Ref  0.99 (0.84- 1.16) | Ref  0.86 | Ref  1.07 (0.91- 1.26) | Ref  0.40 |
| **Having a chronic disease**  No  Yes | Ref  1.06 (0.91- 1.23) | Ref  0.49 | Ref  1.14 (0.98- 1.33) | Ref  0.09 | Ref  1.10 (0.94- 1.28) | Ref  0.23 |
| **Knowing someone with cancer**  No  Yes | Ref  1.57 (1.41- 1.76) | Ref  <0.001 | Ref  1.40 (1.26- 1.57) | Ref  <0.001 | Ref  1.41 (1.26- 1.57) | Ref  <0.001 |
| **Marital status**  Single  Married  Divorced  Widowed | Ref  1.12 (0.95- 1.33)  0.95 (0.61- 1.49)  0.72 (0.48- 1.08) | Ref  0.17  0.83  0.12 | Ref  1.09 (0.92- 1.28)  1.32 (0.84- 2.06)  0.87 (0.58- 1.30) | Ref  0.32  0.23  0.50 | Ref  1.09 (0.92- 1.28)  1.18 (0.75- 1.84)  0.83 (0.55- 1.24) | Ref  0.31  0.48  0.36 |
| **Site of data collection**  Public spaces  Hospitals  Primary healthcare centers | Ref  1.17 (1.01- 1.35)  0.84 (0.73- 0.97) | Ref  0.040  0.017 | Ref  1.13 (0.98- 1.31)  0.79 (0.69- 0.91) | Ref  0.10  0.001 | Ref  1.33 (1.15- 1.54)  0.85 (0.74- 0.98) | Ref  <0.001  0.022 |

AOR= adjusted odds ratio, CI= confidence interval, WBJ= West Bank and Jerusalem.
* Adjusted for age-group, menarche, educational level, occupation, monthly income, residency, having a chronic disease,
knowing someone with cancer, and site of data collection.

| **Characteristic**  Supplementary table 3: Multivariable logistic regression analyzing the association between recognizing other ovarian cancer symptoms and participant characteristics. | **Passing more urine  than usual** | | **Changes in bowel habit** | | **Persistent bloating** | | **Difficulty eating on most days** | | **Feeling full persistently** | |
| --- | --- | --- | --- | --- | --- | --- | --- | --- | --- | --- |
|  | **AOR (95% CI)*** | **p-value** | **AOR (95% CI)*** | **p-value** | **AOR (95% CI)*** | **p-value** | **AOR (95% CI)*** | **p-value** | **AOR (95% CI)*** | **p-value** |
| **Age group**  18 to 44  45 or older | Ref  0.73 (0.62- 0.85) | Ref  <0.001 | Ref  1.06 (0.90- 1.23) | Ref  0.49 | Ref  1.01 (0.86- 1.18) | Ref  0.95 | Ref  1.00 (0.86- 1.18) | Ref  0.97 | Ref  1.04 (0.88- 1.23) | Ref  0.66 |
| **Menarche**  Normal (11-15 years)  Early (≤ 10 years)  Late (≥ 16 years) | Ref  0.83 (0.50- 1.38)  1.38 (1.17- 1.62) | Ref  0.48  <0.001 | Ref  0.93 (0.56- 1.54)  1.04 (0.88- 1.22) | Ref  0.77  0.67 | Ref  0.97 (0.59- 1.61)  1.10 (0.93- 1.30) | Ref  0.92  0.25 | Ref  0.79 (0.46- 1.36)  1.19 (1.01- 1.40) | Ref  0.40  0.044 | Ref  0.87 (0.49- 1.54)  1.04 (0.87- 1.24) | Ref  0.64  0.66 |
| **Educational level**  Illiterate  Primary  Preparatory  Secondary  Diploma  Bachelor  Postgraduate | Ref  1.32 (0.77- 2.29)  1.53 (0.91- 2.58)  2.08 (1.25- 3.48)  1.73 (1.02- 2.95)  2.22 (1.32- 3.73)  2.35 (1.20- 4.60) | Ref  0.31  0.11  0.005  0.043  0.003  0.013 | Ref  1.99 (1.14- 3.49)  1.81 (1.06- 3.09)  2.08 (1.23- 3.54)  2.03 (1.17- 3.51)  2.19 (1.28- 3.75)  2.20 (1.11- 4.38) | Ref  0.016  0.030  0.007  0.012  0.004  0.024 | Ref  1.28 (0.75- 2.18)  1.16 (0.70- 1.92)  1.48 (0.90- 2.43)  1.26 (0.75- 2.13)  1.46 (0.88- 2.42)  2.01 (1.04- 3.89) | Ref  0.37  0.58  0.13  0.38  0.15  0.039 | Ref  1.34 (0.78- 2.30)  1.43 (0.86- 2.39)  1.27 (0.76- 2.10)  1.09 (0.65- 1.85)  1.23 (0.74- 2.06)  0.84 (0.41- 1.70) | Ref  0.28  0.17  0.36  0.74  0.42  0.62 | Ref  0.94 (0.55- 1.62)  1.19 (0.71- 1.98)  0.89 (0.54- 1.48)  0.67 (0.39- 1.15)  0.75 (0.45- 1.25)  0.89 (0.44- 1.80) | Ref  0.83  0.51  0.66  0.14  0.27  0.75 |
| **Occupation**  Housewife  Employed  Retired  Student | Ref  0.79 (0.67- 0.94)  0.90 (0.49- 1.65)  0.94 (0.75- 1.17) | Ref  0.006  0.73  0.57 | Ref  0.88 (0.74- 1.03)  1.36 (0.75- 2.48)  0.92 (0.74- 1.16) | Ref  0.12  0.31  0.50 | Ref  0.93 (0.78- 1.10)  0.90 (0.48- 1.69)  1.15 (0.92- 1.44) | Ref  0.38  0.75  0.23 | Ref  0.92 (0.77- 1.09)  1.11 (0.60- 2.07)  0.81 (0.64- 1.02) | Ref  0.33  0.73  0.07 | Ref  0.90 (0.75- 1.09)  1.32 (0.69- 2.53)  0.80 (0.62- 1.04) | Ref  0.27  0.40  0.10 |
| **Monthly income**  < 1450 NIS  ≥ 1450 NIS | Ref  1.05 (0.89- 1.23) | Ref  0.60 | Ref  1.03 (0.88- 1.22) | Ref  0.69 | Ref  0.88 (0.74- 1.04) | Ref  0.12 | Ref  0.88 (0.75- 1.05) | Ref  0.33 | Ref  1.06 (0.89- 1.27) | Ref  0.50 |
| **Residency**  Gaza Strip  WBJ | Ref  0.96 (0.82- 1.13) | Ref  0.64 | Ref  0.98 (0.83- 1.16) | Ref  0.83 | Ref  0.87 (0.74- 1.02) | Ref  0.09 | Ref  0.78 (0.66- 0.92) | Ref  0.003 | Ref  0.64 (0.53- 0.76) | Ref  <0.001 |
| **Having a chronic disease**  No  Yes | Ref  1.05 (0.90- 1.23) | Ref  0.52 | Ref  1.05 (0.90- 1.22) | Ref  0.54 | Ref  1.07 (0.92- 1.25) | Ref  0.38 | Ref  1.06 (0.90- 1.23) | Ref  0.50 | Ref  1.05 (0.89- 1.24) | Ref  0.57 |
| **Knowing someone with cancer**  No  Yes | Ref  1.38 (1.23- 1.54) | Ref  <0.001 | Ref  1.38 (1.23- 1.54) | Ref  <0.001 | Ref  1.36 (1.22- 1.53) | Ref  <0.001 | Ref  1.26 (1.12- 1.41) | Ref  <0.001 | Ref  1.13 (1.01- 1.28) | Ref  0.047 |
| **Marital status**  Single  Married  Divorced  Widowed | Ref  1.30 (1.10- 1.53)  1.10 (0.70- 1.72)  0.68 (0.44- 1.05) | Ref  0.002  0.69  0.08 | Ref  1.08 (0.91- 1.28)  1.18 (0.75- 1.85)  0.85 (0.56- 1.29) | Ref  0.38  0.48  0.44 | Ref  1.16 (0.98- 1.37)  1.51 (0.97- 2.37)  0.99 (0.66- 1.51) | Ref  0.09  0.07  0.99 | Ref  0.92 (0.77- 1.09)  0.99 (0.63- 1.57)  0.93 (0.62- 1.41) | Ref  0.32  0.97  0.75 | Ref  0.92 (0.76- 1.11)  0.91 (0.54- 1.51)  0.82 (0.52- 1.28) | Ref  0.37  0.71  0.37 |
| **Site of data collection**  Public spaces  Hospitals  Primary healthcare centers | Ref  1.12 (0.97- 1.30)  0.88 (0.76- 1.01) | Ref  0.13  0.06 | Ref  1.36 (1.17- 1.57)  1.06 (0.92- 1.22) | Ref  <0.001  0.45 | Ref  1.14 (0.98- 1.32)  0.85 (0.74- 0.98) | Ref  0.08  0.025 | Ref  1.04 (0.90- 1.21)  0.92 (0.80- 1.07) | Ref  0.59  0.29 | Ref  1.80 (1.53- 2.11)  1.02 (0.87- 1.20) | Ref  <0.001  0.82 |

AOR= adjusted odds ratio, CI= confidence interval, WBJ= West Bank and Jerusalem.
* Adjusted for age-group, menarche, educational level, occupation, monthly income, residency, having a chronic disease,
knowing someone with cancer, and site of data collection.

Supplementary table 4: Bivariable logistic regression analyzing the association between recognizing the three most identified symptoms of ovarian cancer and participant characteristics.

| **Characteristic** | **Extreme generalized fatigue** | | **Unexplained weight loss** | | **Increased abdominal size on most days** | |
| --- | --- | --- | --- | --- | --- | --- |
|  | **COR (95% CI)** | **p-value** | **COR (95% CI)** | **p-value** | **COR (95% CI)** | **p-value** |
| **Age group**  18 to 44  45 or older | Ref  0.87 (0.76- 1.00) | Ref  0.051 | Ref  1.02 (0.89- 1.17) | Ref  0.78 | Ref  0.92 (0.81- 1.04) | Ref  0.18 |
| **Menarche**  Normal (11-15 years)  Early (≤ 10 years)  Late (≥ 16 years) | Ref  0.71 (0.43- 1.17)  0.99 (0.83- 1.18) | Ref  0.18  0.90 | Ref  0.92 (0.55- 1.54)  1.08 (0.91- 1.28) | Ref  0.75  0.38 | Ref  0.78 (0.48- 1.28)  1.10 (0.93- 1.29) | Ref  0.32  0.28 |
| **Educational level**  Illiterate  Primary  Preparatory  Secondary  Diploma  Bachelor  Postgraduate | Ref  1.55 (0.94- 2.56)  2.33 (1.45- 3.75)  2.12 (1.34- 3.36)  1.86 (1.15- 3.01)  2.46 (1.55- 3.90)  3.40 (1.71- 6.76) | Ref  0.09  0.001  0.001  0.012  <0.001  <0.001 | Ref  1.25 (0.75- 2.09)  1.62 (0.99- 2.62)  1.40 (0.88- 2.24)  1.24 (0.76- 2.02)  1.33 (0.83- 2.13)  1.78 (0.92- 3.43) | Ref  0.39  0.051  0.16  0.38  0.23  0.09 | Ref  1.24 (0.75- 2.04)  1.62 (1.01- 2.59)  1.78 (1.12- 2.82)  1.47 (0.91- 2.38)  1.80 (1.14- 2.86)  1.70 (0.91- 3.17) | Ref  0.41  0.046  0.014  0.11  0.012  0.10 |
| **Occupation**  Housewife  Employed  Retired  Student | Ref  0.80 (0.69- 0.92)  0.64 (0.35- 1.16)  1.06 (0.87- 1.29) | Ref  0.002  0.14  0.54 | Ref  0.79 (0.68- 0.90)  0.81 (0.44- 1.47)  0.72 (0.60- 0.86) | Ref  0.001  0.48  <0.001 | Ref  0.88 (0.77- 1.01)  0.74 (0.41- 1.31)  1.03 (0.86- 1.23) | Ref  0.08  0.30  0.75 |
| **Monthly income**  < 1450 NIS  ≥ 1450 NIS | Ref  1.10 (0.98- 1.24) | Ref  0.10 | Ref  0.90 (0.80- 1.02) | Ref  0.09 | Ref  0.98 (0.87- 1.09) | Ref  0.68 |
| **Residency**  Gaza Strip  WBJ | Ref  0.96 (0.85- 1.08) | Ref  0.45 | Ref  0.89 (0.79- 1.00) | Ref  0.051 | Ref  0.84 (0.75- 0.94) | Ref  0.002 |
| **Having a chronic disease**  No  Yes | Ref  1.10 (0.95- 1.28) | Ref  0.20 | Ref  1.06 (0.92- 1.22) | Ref  0.40 | Ref  0.99 (0.86- 1.13) | Ref  0.87 |
| **Knowing someone with cancer**  No  Yes | Ref  1.73 (1.53- 1.94) | Ref  <0.001 | Ref  1.72 (1.53- 1.92) | Ref  <0.001 | Ref  1.44 (1.29- 1.61) | Ref  <0.001 |
| **Marital status**  Single  Married  Divorced  Widowed | Ref  1.25 (1.09- 1.43)  0.98 (0.62- 1.56)  0.73 (0.50- 1.07) | Ref  0.002  0.95  0.11 | Ref  1.43 (1.25- 1.63)  1.14 (0.73- 1.78)  1.30 (0.88- 1.93) | Ref  <0.001  0.57  0.19 | Ref  1.14 (1.01- 1.30)  0.80 (0.52- 1.23)  0.81 (0.56- 1.18) | Ref  0.041  0.30  0.28 |
| **Site of data collection**  Public spaces  Hospitals  Primary healthcare centers | Ref  1.33 (1.14- 1.56)  0.74 (0.64- 0.85) | Ref  <0.001  <0.001 | Ref  1.52 (1.31- 1.76)  0.77 (0.67- 0.88) | Ref  <0.001  <0.001 | Ref  1.37 (1.19- 1.57)  0.88 (0.77- 1.01) | Ref  <0.001  0.06 |

COR= crude odds ratio, CI= confidence interval, WBJ= West Bank and Jerusalem.

Supplementary table 5: Bivariable logistic regression analyzing the association between recognizing ovarian cancer symptoms with pain and participant characteristics.

| **Characteristic** | **Persistent low back pain** | | **Persistent pain in the pelvis** | | **Persistent pain in the abdomen** | |
| --- | --- | --- | --- | --- | --- | --- |
|  | **COR (95% CI)** | **p-value** | **COR (95% CI)** | **p-value** | **COR (95% CI)** | **p-value** |
| **Age group**  18 to 44  45 or older | Ref  0.87 (0.76- 0.98) | Ref  0.027 | Ref  0.99 (0.88- 1.13) | Ref  0.91 | Ref  1.04 (0.92- 1.18) | Ref  0.54 |
| **Menarche**  Normal (11-15 years)  Early (≤ 10 years)  Late (≥ 16 years) | Ref  0.83 (0.51- 1.35)  1.09 (0.93- 1.29) | Ref  0.45  0.29 | Ref  0.84 (0.51- 1.37)  0.90 (0.76- 1.05) | Ref  0.48  0.19 | Ref  1.12 (0.68- 1.82)  1.11 (0.94- 1.30) | Ref  0.66  0.21 |
| **Educational level**  Illiterate  Primary  Preparatory  Secondary  Diploma  Bachelor  Postgraduate | Ref  1.75 (1.05- 2.92)  2.03 (1.25- 3.30)  2.39 (1.48- 3.84)  2.05 (1.25- 3.36)  2.62 (1.63- 4.22)  2.62 (1.39- 4.95) | Ref  0.034  0.004  <0.001  0.004  <0.001  0.003 | Ref  0.92 (0.56- 1.53)  0.98 (0.61- 1.56)  1.10 (0.69- 1.74)  1.05 (0.65- 1.69)  1.30 (0.82- 2.06)  1.87 (1.00- 3.51) | Ref  0.76  0.92  0.69  0.85  0.26  0.051 | Ref  1.20 (0.72- 2.00)  1.47 (0.91- 2.38)  1.42 (0.89- 2.27)  1.37 (0.84- 2.24)  1.51 (0.95- 2.42)  1.85 (0.99- 3.46) | Ref  0.48  0.12  0.14  0.20  0.08  0.06 |
| **Occupation**  Housewife  Employed  Retired  Student | Ref  0.93 (0.82- 1.07)  0.61 (0.34- 1.09)  1.28 (1.07- 1.53) | Ref  0.33  0.09  0.007 | Ref  1.10 (0.96- 1.26)  0.74 (0.42- 1.33)  1.67 (1.40- 2.00) | Ref  0.18  0.32  <0.001 | Ref  0.96 (0.84- 1.10)  0.55 (0.30- 1.00)  1.03 (0.87- 1.22) | Ref  0.59  0.051  0.74 |
| **Monthly income**  < 1450 NIS  ≥ 1450 NIS | Ref  1.04 (0.93- 1.16) | Ref  0.47 | Ref  1.27 (1.13- 1.41) | Ref  <0.001 | Ref  1.05 (0.94- 1.17) | Ref  0.36 |
| **Residency**  Gaza Strip  WBJ | Ref  0.89 (0.80- 0.99) | Ref  0.032 | Ref  1.18 (1.06- 1.31) | Ref  0.003 | Ref  1.07 (0.96- 1.19) | Ref  0.21 |
| **Having a chronic disease**  No  Yes | Ref  0.93 (0.82- 1.07) | Ref  0.32 | Ref  1.06 (0.93- 1.22) | Ref  0.36 | Ref  1.09 (0.96- 1.25) | Ref  0.19 |
| **Knowing someone with cancer**  No  Yes | Ref  1.55 (1.39- 1.73) | Ref  <0.001 | Ref  1.42 (1.27- 1.58) | Ref  <0.001 | Ref  1.42 (1.28- 1.58) | Ref  <0.001 |
| **Marital status**  Single  Married  Divorced  Widowed | Ref  1.00 (0.88- 1.14)  0.81 (0.52- 1.24)  0.58 (0.40- 0.84) | Ref  0.99  0.33  0.004 | Ref  0.85 (0.75- 0.96)  1.03 (0.69- 1.60)  0.71 (0.49- 1.03) | Ref  0.012  0.89  0.07 | Ref  1.06 (0.93- 1.20)  1.11 (0.72- 1.71)  0.84 (0.58- 1.22) | Ref  0.37  0.63  0.37 |
| **Site of data collection**  Public spaces  Hospitals  Primary healthcare centers | Ref  1.02 (0.89- 1.17)  0.77 (0.68- 0.88) | Ref  0.83  <0.001 | Ref  0.96 (0.84- 1.10)  0.69 (0.60- 0.79) | Ref  0.52  <0.001 | Ref  1.24 (1.08- 1.42)  0.80 (0.70- 0.91) | Ref  0.002  0.001 |

COR= crude odds ratio, CI= confidence interval, WBJ= West Bank and Jerusalem.

COR= crude odds ratio, CI= confidence interval, WBJ= West Bank and Jerusalem.

| **Characteristic** | **Passing more urine  than usual** | | **Changes in bowel habit** | | **Persistent bloating** | | **Difficulty eating on most days** | | **Feeling full persistently** | |
| --- | --- | --- | --- | --- | --- | --- | --- | --- | --- | --- |
|  | **COR (95% CI)** | **p-value** | **COR (95% CI)** | **p-value** | **COR (95% CI)** | **p-value** | **COR (95% CI)** | **p-value** | **COR (95% CI)** | **p-value** |
| **Age group**  18 to 44  45 or older | Ref  0.68 (0.60- 0.77) | Ref  <0.001 | Ref  1.09 (0.96- 1.23) | Ref  0.21 | Ref  0.99 (0.87- 1.12) | Ref  0.83 | Ref  1.06 (0.93- 1.20) | Ref  0.42 | Ref  1.19 (1.03- 1.36) | Ref  0.015 |
| **Menarche**  Normal (11-15 years)  Early (≤ 10 years)  Late (≥ 16 years) | Ref  0.84 (0.51- 1.38)  1.38 (1.18- 1.62) | Ref  0.50  <0.001 | Ref  0.97 (0.59- 1.60)  1.05 (0.89- 1.23) | Ref  0.92  0.59 | Ref  1.01 (0.62- 1.67)  1.13 (0.96- 1.33) | Ref  0.96  0.14 | Ref  0.79 (0.47- 1.35)  1.22 (1.03- 1.43) | Ref  0.39  0.018 | Ref  0.89 (0.51- 1.55)  1.10 (0.92- 1.30) | Ref  0.68  0.31 |
| **Educational level**  Illiterate  Primary  Preparatory  Secondary  Diploma  Bachelor  Postgraduate | Ref  1.43 (0.84- 2.45)  1.87 (1.12- 3.11)  2.49 (1.51- 4.10)  1.80 (1.07- 3.02)  2.32 (1.41- 3.82)  2.20 (1.15- 4.20) | Ref  0.19  0.016  <0.001  0.026  0.001  0.017 | Ref  2.17 (1.24- 3.78)  1.93 (1.14- 3.28)  1.99 (1.18- 3.34)  1.82 (1.07- 3.12)  1.91 (1.13- 3.21)  1.92 (0.99- 3.75) | Ref  0.006  0.015  0.009  0.028  0.015  0.06 | Ref  1.37 (0.81- 2.32)  1.25 (0.76- 2.06)  1.51 (0.93- 2.47)  1.16 (0.70- 1.92)  1.33 (0.82- 2.17)  1.69 (0.89- 3.21) | Ref  0.24  0.38  0.10  0.58  0.25  0.11 | Ref  1.38 (0.81- 2.36)  1.50 (0.91- 2.49)  1.30 (0.79- 2.13)  1.06 (0.63- 1.76)  1.15 (0.70- 1.89)  0.74 (0.38- 1.48) | Ref  0.24  0.11  0.30  0.84  0.57  0.40 | Ref  1.01 (0.59- 1.71)  1.27 (0.77- 2.09)  0.87 (0.53- 1.41)  0.60 (0.36- 1.00)  0.66 (0.40- 1.07)  0.74 (0.38- 1.46) | Ref  0.98  0.36  0.56  0.052  0.09  0.39 |
| **Occupation**  Housewife  Employed  Retired  Student | Ref  0.79 (0.69- 0.91)  0.68 (0.38- 1.23)  0.93 (0.78- 1.11) | Ref  0.001  0.21  0.42 | Ref  0.86 (0.75- 0.99)  1.45 (0.81- 2.57)  0.84 (0.70- 0.99) | Ref  0.032  0.21  0.049 | Ref  0.84 (0.73- 0.97)  0.82 (0.45- 1.49)  1.00 (0.84- 1.20) | Ref  0.016  0.52  0.97 | Ref  0.78 (0.68- 0.90)  0.91 (0.50- 1.66)  0.76 (0.63- 0.91) | Ref  0.001  0.76  0.003 | Ref  0.67 (0.58- 0.79)  1.03 (0.55- 1.90)  0.60 (0.49- 0.74) | Ref  <0.001  0.93  <0.001 |
| **Monthly income**  < 1450 NIS  ≥ 1450 NIS | Ref  0.97 (0.87- 1.09) | Ref  0.63 | Ref  1.00 (0.90- 1.12) | Ref  0.94 | Ref  0.81 (0.72- 0.90) | Ref  <0.001 | Ref  0.72 (0.64- 0.80) | Ref  0.33 | Ref  0.73 (0.65- 0.82) | Ref  <0.001 |
| **Residency**  Gaza Strip  WBJ | Ref  0.90 (0.81- 1.00) | Ref  0.051 | Ref  1.00 (0.89- 1.11) | Ref  0.94 | Ref  0.80 (0.72- 0.90) | Ref  <0.001 | Ref  0.72 (0.64- 0.80) | Ref  <0.001 | Ref  0.69 (0.61- 0.78) | Ref  <0.001 |
| **Having a chronic disease**  No  Yes | Ref  0.86 (0.76- 0.99) | Ref  0.031 | Ref  1.10 (0.96- 1.26) | Ref  0.15 | Ref  1.04 (0.91- 1.19) | Ref  0.54 | Ref  1.09 (0.95- 1.25) | Ref  0.22 | Ref  1.19 (1.03- 1.38) | Ref  0.016 |
| **Knowing someone with cancer**  No  Yes | Ref  1.35 (1.21- 1.51) | Ref  <0.001 | Ref  1.37 (1.23- 1.53) | Ref  <0.001 | Ref  1.37 (1.23- 1.53) | Ref  <0.001 | Ref  1.27 (1.13- 1.41) | Ref  <0.001 | Ref  1.15 (1.02- 1.30) | Ref  0.019 |
| **Marital status**  Single  Married  Divorced  Widowed | Ref  1.23 (1.09- 1.40)  0.93 (0.60- 1.44)  0.49 (0.33- 0.73) | Ref  0.001  0.76  0.001 | Ref  1.19 (1.04- 1.35)  1.20 (0.77- 1.86)  0.95 (0.65- 1.40) | Ref  0.011  0.42  0.80 | Ref  1.14 (1.00- 1.30)  1.44 (0.94- 2.23)  0.99 (0.67- 1.45) | Ref  0.06  0.10  0.95 | Ref  1.14 (0.99- 1.30)  1.16 (0.74- 1.81)  1.19 (0.81- 1.75) | Ref  0.06  0.52  0.37 | Ref  1.35 (1.16- 1.56)  1.10 (0.67- 1.81)  1.29 (0.86- 1.94) | Ref  <0.001  0.70  0.22 |
| **Site of data collection**  Public spaces  Hospitals  Primary healthcare centers  COR= crude odds ratio, CI= confidence interval, WBJ= West Bank and Jerusalem. | Ref  1.08 (0.94- 1.23)  0.88 (0.78- 1.01) | Ref  0.28  0.06 | Ref  1.33 (1.16- 1.53)  1.03 (0.90- 1.17) | Ref  <0.001  0.69 | Ref  1.10 (0.96- 1.26)  0.84 (0.74- 0.96) | Ref  0.18  0.011 | Ref  1.11 (0.96- 1.27)  0.97 (0.85- 1.11) | Ref  0.16  0.67 | Ref  1.98 (1.70- 2.30)  1.10 (0.94- 1.28) | Ref  <0.001  0.22 |

Supplementary table 6: Bivariable logistic regression analyzing the association between recognizing other ovarian cancer symptoms and participant characteristics.
